# Association between VA providers’ reported use of care strategies and emergence delirium: a national retrospective cross-sectional survey

**DOI:** 10.1186/s13741-026-00700-6

**Published:** 2026-05-21

**Authors:** Monique Y. Boudreaux-Kelly, Matthew A. Taylor, William M. Pileggi, Michael J. Boland, David V. Julian, Amanda K. Beckstead

**Affiliations:** 1 VA Pittsburgh Medical Center – University Drive, University Drive C, Pittsburgh, PA 15240-1003 USA; 2 Pennsylvania Patient Safety Authority, 333 Market Street, Lobby Level, Harrisburg, PA 17101 USA; 3 UPMC Altoona Medical Center, 620 Howard Ave, Altoona, PA 16601 USA

**Keywords:** General anesthesia, Monitored anesthesia care, Post-traumatic stress disorder, Military Veteran, Agitation, Anesthetic medication, Screening

## Abstract

**Background:**

Emergence delirium (ED) is a short-term behavioral condition associated with risk of harm that may occur when a patient awakens from anesthesia. A national survey was conducted to evaluate our hypothesis that Veterans Health Administration (VHA) anesthesia providers who reported routinely screening patients for ED risk and selecting medication strategies associated with lower ED risk would be less likely to report estimated ED occurrence.

**Methods:**

A retrospective cross-sectional self-report survey was designed by staff at Veterans Affairs’ Pittsburgh Healthcare System (PHS) and launched nationally to VHA anesthesia providers. The survey was conducted after an intervention at PHS to increase routine patient screening for ED risk and use of the “*exact* medication” strategy promoted by the intervention regardless of location: administration of propofol, dexmedetomidine, and ketamine and avoidance of midazolam and volatile anesthetics. Comparisons of percentage of PHS and non-PHS anesthesia providers who reported use of the “*exact* medication” strategy and estimated ED occurrence were analyzed using tests of independence and multivariable logistic regression models.

**Results:**

Based on survey data from 690 VHA anesthesia providers (PHS *n* = 34, non-PHS *n* = 656), both routine screening (PHS: 88.2%, non-PHS: 58.4%, *p* < 0.001) and the “e*xact* medication” strategy (PHS: 44.1%, non-PHS: 1.4%, *p* < 0.001) were reported by more PHS than non-PHS providers. Survey results indicated 37.3% more providers at PHS (38.2%, *n* = 13) reported using the “routine screening plus *exact* medication” strategy than non-PHS (0.9%, *n* = 6, *p* < 0.001) and providers who used that care strategy were less likely to report estimated ED occurrence (PHS: 46% vs non-PHS: 50%, *p* < 0.05). Using multivariable modeling analysis, the “routine screening plus *exact* medication” care strategy was associated with lower odds of estimated ED occurrence regardless of location (OR = 0.36, *p* < 0.05).

**Conclusions:**

Bivariate comparisons and multivariable adjusted modeling of survey findings supported our hypothesis that providers who reported routinely screening patients for ED risk and employing the "*exact* medication" strategy were less likely to have estimated ED occurrence. Use of these descriptive analyses to generate hypotheses for future assessments is recommended.

## Background

Emergence delirium (ED) (i.e., agitation or excitation) is a transient behavioral condition that may occur when a patient awakens from an anesthetic and/or adjunct agent (Cole et al. 2002; S.-J. Lee and Sung 2020; Lepouse et al. 2006; Taylor and Pileggi 2021; Taylor et al. 2022b; Tolly et al. 2021; Urits et al. 2020). ED may elicit harmful patient behaviors, including thrashing, kicking, punching, attempting to exit the bed/table, and hallucinations (Cole et al. 2002; S.-J. Lee and Sung 2020; Taylor and Pileggi 2021; Taylor et al. 2022b; Tolly et al. 2021; Urits et al. 2020). In one study, 70% of ED-associated safety event reports described patients engaging in “dangerous” behavior(s) and there was a statistically significant relation with injuries (Taylor and Pileggi 2021). The same study estimated that “…54% of [the ED] events described patient behavior that created an immediate and high risk of harm for the staff” (Taylor and Pileggi 2021).

Several studies of ED have explored medication and screening care strategies used by anesthesia providers intending to prevent and/or treat occurrence of ED in adults (Bartoszek et al. 2021; Huang et al. 2023; Taylor et al. 2022b; Yuan et al. 2022). A comprehensive list of studies we previously identified that explored care strategies for ED is available in Supplemental Material S6 of our related publication (Taylor et al. 2022b). However, few have assessed the effect of multiple concurrent medications plus screening care strategies on risk of ED while using a large national sample of providers who regularly care for adults at high risk of ED. Even among ED studies focused solely on medications (Taylor et al. 2022b), the evaluations of medications possibly related to ED, which include midazolam, dexmedetomidine, ketamine, volatile anesthetics, and propofol, have produced mixed, sometimes conflicting, results indicating a need for studies of the effect of multiple medications measured concurrently on occurrence of ED, especially among patients who may have higher risk and greater vulnerability to ED.

The Veterans Health Administration (VHA) is one of the largest integrated health care networks in the United States (Veterans Health Administration, 2022). Despite the ability to conduct large studies of ED at the Department of Veterans Affairs’ (VA) and that Veterans may be considered at high-risk for ED due to the prevalence of various risk factors, such as post-traumatic stress disorder (PTSD), (Bartoszek et al. 2021; Beckstead et al. 2020; McGuire 2012; Tolly et al. 2021; Umholtz et al. 2016) there are few national studies of prevalence and prevention of ED at the VA. Based upon the precedent of using staff surveys in adult ED research, (Bustos & Sambuca, 2022; Heily et al. 2022; Maasdam, 2020; John Tyler Wilson 2013, 2014; J T Wilson and Pokorny 2012; Yuan et al. 2022) we designed a national VA-wide retrospective cross-sectional survey for VA anesthesia providers that allowed us to measure the percentage of providers who estimated ED occurring at least once per month in their patients and the care strategies they reported providing to patients at high-risk for developing ED. The survey was designed to compare findings from VA anesthesia providers across two mutually exclusive locations; Veterans Affairs’ Pittsburgh Healthcare System (PHS), where staff previously designed and implemented an intervention to prevent and treat ED, (Taylor et al. 2022b) and all other VA facilities across the VA national care network (non-PHS). The four aims of our survey study were to: (1) assess location differences in providers’ reported use of routine screening and medication strategies (PHS versus non-PHS); (2) evaluate location differences in percentage of providers who estimated ED (PHS versus non-PHS); (3) measure associations, regardless of location, between percentage of providers who estimated ED and reported use of routine screening and medication strategies; and (4) measure differences in percentage of providers who estimated ED between PHS and non-PHS locations while adjusting for other relevant factors, including reported use of routine screening and medications. We hypothesized that PHS providers would have lower percentage of providers who estimated ED occurrence compared to providers at non-PHS facilities and the care strategies promoted at PHS would be associated with lower percentage of providers who estimated ED regardless of location.

## Methods

We utilized a cross-sectional survey of national VHA anesthesia providers to assess our aim by requesting estimates of ED occurrences they observed per month in 2021 and the care strategies they reported providing to patients at high-risk for developing ED, specifically patients with PTSD.

### Setting, patients, and staff 

VHA consists of 171 medical centers and serves approximately 9 million Veterans per year.(U.S. Department of Veterans Affairs, 2022; Veterans Health Administration, 2022) Based on a patient sample from a recent national study, VHA patients had an average age of 57.5 years, were 88.5% male, 46.8% had a substance use disorder, 40.2% received mental health treatment, 31.0% had chronic pain, 30.1% had a sleep disorder, 21.8% had PTSD, and 0.7% had a traumatic brain injury.(Meffert et al. 2019) Given the rates of diagnoses and conditions among Veteran patients and findings from previous studies of risk factors for ED, (Beckstead et al. 2020; S.-J. Lee and Sung 2020; McGuire 2012; McGuire and Burkard 2010; Shen et al. 2023; Taylor and Pileggi 2021; Tolly et al. 2021; Viswanath et al. 2015) the Veteran population is at higher risk for ED than the general population.

PHS’ Level I medical center has 146 acute care beds (VA Pittsburgh Healthcare System, 2022) and, during 2020, completed more than 9,078 procedures that involved anesthesia (e.g., operating rooms, gastrointestinal, electro-physiology). The anesthesia department at PHS has a total of 43 anesthesia providers with 11 physician anesthesiologists and 32 certified registered nurse anesthetists (CRNAs). The anesthesia department uses a physician and CRNA team model, where the attending physician oversees up to 4 procedures with 1 CRNA per procedure.

### Brief description of the intervention used at PHS 

Prior to 2018, PHS had ED-related issues with patient and staff safety. In 2018, staff at PHS designed and implemented an intervention with the goal of decreasing the rate and severity of ED and improving patient and staff safety at PHS (Table 1) (Pileggi et al. 2023; Taylor et al. 2022a, b). The intervention included patient evaluation, individualized combinations of care components, and spanned across the perioperative phases. Care components that were individualized were routine screening for risk factors, enhancing communication among staff, adjusting the environment, following a medication strategy, and training staff to safely apply manual hands-on restraint to reduce the likelihood of patient injury. For patients identified as moderate to high risk for ED according to routine screening, the intervention recommended avoiding use of midazolam and volatile anesthetics, and, as an alternative, administering propofol, dexmedetomidine, and ketamine (Taylor et al. 2022a, b). The intervention was implemented at PHS in June 2018 and involved anesthesia providers and other perioperative staff, including but not limited to nurses who specialized in operating room, gastrointestinal, preoperative, and post-anesthesia care unit (PACU) care, surgical technicians, medical residents, and student CRNAs. For more information about the intervention and literature that guided development of the medication strategy including rates and doses, see our previously published articles (Taylor et al. 2022a, b) and our open access training video (Pileggi et al. 2023).

**Table 1:** PHS Intervention steps: primary clinical components across perioperative phases

Component 1	Conduct a preoperative anesthesia screening to identify patients at an elevated risk for ED and gather information to orient the patient upon emergence.
Component 2	Enhance communication with surgical team around patient risk for ED (e.g., call ahead to procedure room and post-anesthesia care unit to initiate intervention protocol, during time out discuss risk of ED and inform staff of IV location and type of airway, proactively request additional staff for support during emergence).
Component 3	For patients identified as moderate to high risk for ED, avoid use of midazolam and volatile anesthetics, and, as an alternative, administer propofol, dexmedetomidine, and ketamine.
Component 4	Adjust immediate post-anesthesia environment to reduce stimulation of patient (e.g., dim lights, minimize noise, place in low traffic area) and mitigate the risk of harm (e.g., adjust table or bed and secure the IV).
Component 5	Attempt to orient the patient by stating familiar people and places (e.g., patient name, partner name, location of hospital).
Component 6	If needed, apply specific hands-on manual restraint techniques to prevent a patient who is engaging in dangerous behavior from harming themself and staff.
Component 7	Following an episode of ED, conduct debrief meetings and add a detailed note to the patient’s record.

### Cross-sectional survey of anesthesia providers 

Because there were no existing, systematically collected data sources at the VA by which to objectively measure occurrence of ED and related care practices, we created a novel cross-sectional self-report survey for PHS and non-PHS VA anesthesia providers which would allow us to compare estimations of occurrence of ED and ED-related care strategies. The survey data were collected and analyzed with the purpose of allowing indirect evaluation of the effect of the PHS-specific intervention on estimated ED occurrence and care strategies. The survey was newly developed for this assessment and underwent pre-testing review by two anesthesia experts associated with the current work (W.P., A.B.) plus two additional anesthesia providers at PHS to ensure that the questions were clearly written, and the answer options were appropriate and clinically meaningful. Feedback obtained from pre-testing review was utilized to alter and finalize content and language for the final survey. The survey was not piloted prior to launching. When the survey was launched to PHS staff, we asked all available staff to complete the survey regardless of prior exposure to earlier versions.

Over a ten-week period (August 24, 2021 to November 1, 2021), links to voluntary anonymous surveys were emailed to all anesthesia providers at VA facilities in the United States and its territories. Survey links were sent three separate times to the entire provider email list during those 10 weeks to prompt non-responders to complete the survey. Non-PHS CRNAs or physicians who provided anesthesia were identified from national staff tables located in the VHA Corporate Data Warehouses as those with active email accounts in the 365 days prior to August 2, 2021. Clean lists of PHS providers’ emails were provided by one of the authors (W.P.). To ensure unambiguous comparison of groups by provider location, PHS and non-PHS providers were emailed two different URL links via Alchemer.com for completion of the same survey. We did not perform an a priori sample size estimation due to an absence of sufficient data sources. Instead, we emailed a survey link to all known anesthesia providers at the VA rather than randomly selecting a representative sample so that we could maximize response rates. No incentives were available for respondents.

### Data collected 

The survey questions, listed in Table 2, focused on assessing providers’ experiences with ED and use of care strategies for managing and preventing ED. The questions targeted the following specific practices promoted by the PHS intervention: conducting a preoperative screening for identifying patients at high risk for ED (Table 1, Component 1) and avoiding use of midazolam and volatile anesthetics while choosing to use instead propofol, dexmedetomidine, and ketamine (Table 1, Component 3). To ensure that we narrowed down to appropriate providers from among those who completed the survey, the survey began with two screening questions confirming that (A) the provider was functioning as a CRNA or physician anesthesiologist in their current position and (B) they had provided anesthesiology care at VHA since January 2021 (i.e., for a minimum of an 8-month period). If the provider selected ‘No’ to either screening question, then the survey ended and no further responses were collected. The survey question topics were estimates of number of surgeries and number of occurrences of ED per month in 2021, U.S. state or territory in which the respondent provided care in 2021, VA facility of care, and individual care strategies such as screening for ED for all patients and medications used in patients with known PTSD (Table 2).

**Table 2:** Survey questions and response options

Survey Questions	Response Options
1. “Are you a CRNA or physician anesthesiologist?”	Selected “yes” or “no”
2. “Have you provided anesthesia services at a Veterans Affairs (VA) facility since January 2021?”	Selected “yes” or “no”
3. “Please select your state.”	Selected U.S. state or territory by drop down menu
4. “Please estimate, on average, how many surgical cases requiring anesthesia you were involved with per month during 2021.”	Entered a whole number
5. “Please estimate, on average, how many instances of emergence delirium/agitation occurred with your patients per month during 2021.”	Entered a whole number
6. “Are your patients routinely pre-screened to identify those at high risk for emergence delirium/agitation? For example, gender or other diagnoses.”	Selected “yes” or “no”
7. “Which of the following drugs do you administer to patients with post-traumatic stress disorder?”	Indicated by a checked box next to each of the following medications: midazolam, volatile anesthetics, dexmedetomidine, propofol, and ketamine

ED was defined for providers as “The condition often begins with awakening from the sedative and/or analgesic agent and is followed by a return to baseline behavior after a short time-period. During the condition, the patient may engage in the following behaviors: violence (e.g., punching, kicking, hitting, biting), thrashing movement, aggression, combativeness, screaming, disconnect with current time and place (e.g., flashbacks, hallucinating, disoriented), attempting to exit bed/table, and/or attempting to remove medical apparatuses.” Because existing PTSD and use of specific medications may separately be associated with risk of ED in patients, the survey inquired about which medications were administered to only patients with PTSD as a measure for broader practice patterns among patients at possible high risk for ED. We did not ask about dosing or timing of medication.

### Variables created from survey data 

Using the reported per month estimated ED number from the survey, we identified which providers reported observing at least one occurrence of ED per month on average and flagged them as ‘Yes’ or ‘No’ for being a provider in whom “estimated ED occurred”. Using the flag variable, we calculated the percentage of providers in whom estimated ED occurred. In these analyses we compared the differences in the percentage estimated ED across PHS and non-PHS locations and in relation to reported care strategies. Based on the survey data collected, we derived the variables used in the statistical analyses (Table 3), including the combination of care strategy definitions of “*similar*” and “*exact* medication” coined in relation to the practices recommended by the PHS intervention (Table 1, Component 3). Medication use was considered *“similar”* if they administered *at least one* of propofol, dexmedetomidine, or ketamine and avoided both midazolam and volatile anesthetics. Medication use was considered *“exact”* if they administered all three of propofol, dexmedetomidine, and ketamine and avoided both midazolam and volatile anesthetics. The variable for location of care was defined by the survey link they completed and was recorded as PHS or non-PHS. Providers who completed the non-PHS survey link and reported being at or had an IP address automatically collected by Alchemer.com indicating they were in Pittsburgh, PA were excluded (n = 8). A total of 10 care strategy variables, estimated ED, and location were created for analysis.

**Table 3:** Summary of analytic variables created from survey data and their definitions and response categories

Analytic Variables Created from Survey Data	Table 2 Question Source and Variable Definitions	Variable Response Categories
Estimated Emergence Delirium (ED)	Question 5: Provider estimated an average monthly occurrence(s) of ED > 0	Yes or no
Location of care	Providers completed copies of the same survey sent via two different links: one for PHS providers and one for non-PHS providers. Link used determined location.	PHS or non-PHS
Individual care strategies • Routine screening • Administration of *midazolam* • Administration of *volatile anesthetics* • Administration of *dexmedetomidine* • Administration of *propofol* • Administration of *ketamine*	Question 6: Routine screening was self-reported as “yes” or “no.” Question 7: Medication use was self-reported by checkboxes.	Yes or no, for each
Combinations of care strategies • *“Similar* medication” strategy: administered *at least one* of ketamine, dexmedetomidine, or propofol; and *did not* administer both midazolam and volatile anesthetics • “Routine screening plus *similar* medication” strategy • *“Exact* medication” strategy: administered ketamine, dexmedetomidine, and propofol; and *did not* administer both midazolam and volatile anesthetics • “Routine screening plus *exact* medication” strategy	Questions 6 and 7: Four variables were created that consisted of different combinations of routine screening and medication use related to the PHS intervention’s recommended medication strategies. The *“similar* medication” strategy variable reflected a medication strategy that *partially* consistent with the strategy promoted at PHS. The “*exact* medication” strategy variable was *completely* consistent with the medication strategy promoted at PHS. The variables that included routine screening indicated providers’ combined responses to self-reported use of routine screening for risk factors associated with ED plus use of “*similar*” or “*exact*” medication strategy.	Yes or no, for each

### Statistical analysis

Because of the cross-sectional survey design, the analytic results were interpreted as associations, not as predictive, causal, or time-based relationships. Only survey data from providers who responded affirmatively to the two screening questions and fully completed the survey were included in these analyses. Survey data were also excluded when a provider entered uninterpretable information, such as random words or symbols, when the IP addresses indicated that completion of the non-PHS survey was done by someone in the city of Pittsburgh, which was considered errant because PHS providers did not have access to the non-PHS provider URL link, or when estimates were too small to expect providers to have had the chance for ED occurrence (< 10 surgeries per month) or excessively large (> 200 surgeries per month or > 50% of cases involved ED). Excessively large estimate exclusion rules were applied knowing that it is common for a physician to oversee multiple CRNAs during their shift. As a result, those physicians’ data could not be separated into the practices of individuals working under them. Because the survey was distributed to all appropriate VHA providers instead of a sample of providers, we did not conduct a priori power analyses. However, the data analysis plan was created a priori. Using the estimated ED flag variable which identified the providers who observed ED in their patients, we calculated and compared the percentage of providers who estimated ED occurrence. Specifically, we evaluated the differences in the percentage estimated ED at PHS and non-PHS locations and in relation to care strategies providers reported using.

Means and standard deviations were calculated for the estimated number of surgeries per month in 2021 and were compared between PHS and non-PHS providers using Wilcoxon rank sum tests. Categorical variables were analyzed for bivariate associations using two-tailed Chi-square or Fisher’s Exact tests of independence, as appropriate, and were quantified using frequencies, percentages, risk differences (RD), and odds ratios (OR) with 95% Wald confidence intervals (CI). Percentages were calculated using cell frequencies divided by column totals (Table 4). RD was selected as a measure of absolute risk between groups to enhance and support the interpretation of relative risk provided by the more standard OR measure (Stegenga, 2015). RD was calculated by subtracting the percentages between cells in the same row under the same headings. RD values for ‘Location’ were interpreted from the perspective of PHS providers, meaning that when more non-PHS providers reported an occurrence it resulted in positive RD. Similarly, a positive RD under the “Estimated Emergence Delirium” heading indicated that a care strategy was associated with greater percentage estimated ED.

**Table 4:** Bivariate group comparisons of reported care strategies and percentage estimated emergence delirium occurrence: location, screening, and medication

Care Strategies and Estimated Emergence Delirium		Location					Percentage Estimated Emergence Delirium (ED)				
		PHS (*n* = 34); %(n)	Non-PHS (*n* = 656); % (*n*)	Odds Ratio (95% CI)	Risk Difference (%)	Location *p*-value	Occurrence: Yes (*n* = 497); %(n)	Occurrence: No (*n* = 193); %(n)	Odds Ratio (95% CI)	Risk Difference (%)	ED *p*-value
Routine Screening for Risk of Emergence Delirium: Yes		88.2% (*n* = 30)	58.4% (*n* = 383)	0.19 (0.07, 0.54)	-29.8%	< 0.001	61.6% (*n* = 306)	55.4% (*n* = 107)	1.29 (0.92, 1.80)	6.2%	0.14
Medications Administered to Patients with PTSD	Midazolam	2.9% (*n* = 1)	64.6% (*n* = 424)	60.31 (8.20, 443.79)	61.7%	< 0.001	61.4% (*n* = 305)	62.2% (*n* = 120)	0.97 (0.69, 1.36)	-0.8%	0.84
	Volatile Anesthetics	38.2% (*n* = 13)	73.5% (*n* = 482)	4.48 (2.22, 9.36)	35.3%	< 0.001	72.3% (*n* = 358)	71.3% (*n* = 137)	1.05 (0.73, 1.52)	1.0%	0.78
	Ketamine	79.4% (*n* = 27)	44.5% (*n* = 292)	0.21 (0.08, 0.46)	-34.9%	< 0.001	46.3% (*n* = 230)	46.1% (*n* = 89)	1.01 (0.72, 1.41)	0.1%	0.96
	Dexmedetomidine	97.1% (*n* = 33)	73.3% (*n* = 481)	0.08 (0.01, 0.61)	-23.8%	< 0.001	76.9% (*n* = 382)	68.4% (*n* = 132)	1.54 (1.06, 2.22)	8.5%	0.02
	Propofol	91.2% (*n* = 31)	83.5% (*n* = 548)	0.49 (0.15, 1.64)	-7.7%	0.24	84.5% (*n* = 420)	82.4% (*n* = 159)	1.17 (0.75, 1.82)	2.1%	0.50
*“Similar* Medication” Strategy: Yes		58.8% (*n* = 20)	14.9% (*n* = 98)	0.12 (0.06, 0.25)	-43.9%	< 0.001	16.9% (*n* = 84)	17.6% (*n* = 34)	0.95 (0.61, 1.47)	-0.7%	0.82
“Routine Screening plus *Similar* Medication” Strategy: Yes		52.9% (*n* = 18)	9.3% (*n* = 61)	0.09 (0.04, 0.19)	-43.6%	< 0.001	11.3% (*n* = 56)	11.9% (*n* = 23)	0.94 (0.56, 1.57)	-0.6%	0.81
“*Exact* Medication” Strategy: Yes		44.1% (*n* = 15)	1.4% (*n* = 9)	0.02 (0.01, 0.04)	-42.7%	< 0.001	2.4% (*n* = 12)	6.2% (*n* = 12)	0.37 (0.16, 0.84)	-3.8%	0.04
“Routine Screening plus *Exact* Medication” Strategy: Yes		38.2% (*n* = 13)	0.9% (*n* = 6)	0.02 (0.005, 0.04)	-37.3%	< 0.001	1.8% (*n* = 9)	5.2% (*n* = 10)	0.34 (0.14, 0.84)	-3.4%	0.02
Estimated Emergence Delirium Occurred: Yes		50.0% (*n* = 17)	73.2% (*n* = 480)	2.73 (1.36, 5.46)	23.2%	0.005	NA	NA	NA	NA	NA

In each column, the percentages were calculated with the numerator from the same cell and the denominator in the column title. Odds Ratio (OR) values indicate the relative odds of reported use of Care Strategy per ‘Location’ and ‘Percentage Estimated ED.’ For ‘Location’ comparisons an OR value over 1.0 means that the non-PHS group has greater odds of that care strategy compared to the PHS group and vice versa for an OR value under 1.0. When the 95% Wald confidence interval (CI) around the OR does not include an OR of 1.00, that means the association with reported use of that care strategy is statistically significant. In the columns labeled “Risk Difference (%)” the percentage in each cell is based on the percentage in the left-hand column minus the percentage in the right-hand column of the same row, under the same heading. NA = not analyzed

In bivariate comparisons, an OR greater than 1.0 indicates a positive association between the variables being evaluated, meaning increases (or decreases) in one variable are associated with increases (or decreases) in the other variable, while a value less than 1.0 means that the increases (or decreases) in one variable are associated with decreases (or increases) in the other variable. For comparison of ‘Location’ and understanding that the reference group is providers at PHS locations, an OR value over 1.00 means there were greater odds of non-PHS providers who reported using that care strategy, such as use of midazolam, compared to PHS providers. If the 95% CI around the OR did not include 1.0, then the association was statistically significant.

Exploratory multivariable logistic regression models were designed to assess the same associations tested using bivariate tests of independence, but while simultaneously adjusting for multiple, possibly confounding, variables. Ten separate models were designed, one for each care strategy variable, using all available data (*N* = 690). In multivariable logistic regression modeling comparisons, two adjusted model versions were designed: (1) version #1 was designed to assess the association of location with estimated ED occurrence after adjusting for care strategy and number of surgeries per month and (2) version #2 was designed to assess the association of care strategy with estimated ED occurrence after adjusting for number of surgeries per month to evaluate overall relationships between care strategies and estimated ED occurrence regardless of location. For version #1, OR values indicated the relative odds of estimated ED occurrence in association with location after adjusting for care strategy and number of surgeries per month. An OR greater than 1.0 for ‘Location’ and both covariates in the ‘Routine Screening for Risk of Emergence Delirium’ model, for example, would indicate that location and both covariates were associated with greater estimated ED occurrence in the non-reference group. Specifically, an OR value greater than 1.0 for ‘Location’ would indicate that non-PHS providers would have greater odds of estimated ED occurrence than PHS providers, for ‘Care Strategy’ would indicate that reported use of ‘Routine Screening for Risk of Emergence Delirium’ would be associated with greater odds of estimated ED occurrence than not having reported use of routine screening, and for ‘Number of Surgeries’ that the higher the number estimated the greater the odds of having estimated ED occurrence. For version #2, OR values indicated the relative odds of estimated ED occurrence in association with each ‘Care Strategy’ after adjusting for number of surgeries per month and irrespective of location. Specifically, an OR value greater than 1.0 for ‘Care Strategy’ would indicate that reported use of ‘Routine Screening for Risk of Emergence Delirium’ would be associated with greater odds of estimated ED occurrence than not having reported use of routine screening and for ‘Number of Surgeries’ that the higher the number estimated the greater the odds of having estimated ED occurrence.

Two-tailed p-values are presented for all statistical comparisons, and statistical significance was set at *p* < 0.05. NA in the tables indicates that analyses were not conducted, as appropriate. Analyses were conducted using SAS software, Version 9.4 (Copyright © 2016 by SAS Institute Inc., Cary, NC, USA.). This work was determined by the Veteran Affairs’ PHS Institutional Review Board (IRB) to be operational activity and exempt from IRB overview. The authors reported no conflicts of interest. Data are available upon request.

## Results

Of the 4,331 potential VHA providers who were emailed a survey link, 633 providers were either contacted in error (*n* = 66) or had non-working emails (*n* = 567). From the eligible remaining providers (*n* = 3,698), 884 providers from 50 states and 1 US territory completed the survey, resulting in a 24% response rate. After exclusions were applied, a total of 656 non-PHS and 34 PHS providers completed the survey and were included in the analyses (*N* = 690). Respondents took an average of 3 min to complete the survey.

### Unadjusted analyses of location (Table 4)

The percentage of providers who reported routinely screening patients for risk of ED was 29.8% lower among non-PHS providers than PHS providers (RD = -29.8%, OR (95% CI) = 0.19 (0.07, 0.54), p < 0.001). For patients with PTSD, significantly more providers at non-PHS reported administering midazolam by 61.7% (RD = 61.7%, OR (95% CI) = 60.31 (8.20, 443.79), p < 0.001) and volatile anesthetics by 35.3% (RD = 35.3%, OR (95% CI) = 4.48 (2.22, 9.36), p < 0.001) than PHS providers. In addition, significantly fewer providers at non-PHS locations reported using ketamine (RD = -34.9%, OR (95% CI) = 0.21 (0.08, 0.46), p < 0.001) and dexmedetomidine (RD = -23.8%, OR (95% CI) = 0.08 (0.01, 0.61), p = 0.002) compared to PHS providers. Among combined care strategy measures, non-PHS providers reported using both the *“similar* medication” (RD = -43.9%, OR (95% CI) = 0.12 (0.06, 0.25), *p* < 0.001) and the “routine screening plus *similar* medication” (RD = -43.6%, OR (95% CI) = 0.09 (0.04, 0.19), *p* < 0.001) strategies significantly less than PHS providers. Significantly fewer non-PHS providers reported using both “*exact* medication” (RD= -42.7%, OR (95% CI) = 0.02 (0.01, 0.04)) and “routine screening plus *exact* medication” (RD= -37.3%, OR (95% CI) = 0.02 (0.005, 0.04)) strategies than PHS providers (*p* < 0.001 for both strategies). Location differences in reported percentage estimated ED were found such that, despite PHS and non-PHS providers having similar numbers of surgeries with anesthesia per month (PHS: mean = 43.64, SD = 24.94; non-PHS: mean = 53.53, SD = 33.52, *p* = 0.06) *[Data not shown in table]*, non-PHS providers were 23.2% more likely to have estimated ED occurrence than PHS providers (RD = 23.2%, OR (95% CI) = 2.73 (1.36, 5.46), *p* = 0.005).

### Unadjusted analyses of percentage estimated ED (Table 4)

Separate percentage estimated ED analyses measured the associations between providers who estimated ED occurrence and care strategies they reported utilizing regardless of location, meaning that data from all eligible respondents were combined for comparisons. Routine screening was not associated with differences of estimated ED occurrence (RD = 6.2%, OR (95% CI) = 1.29 (0.92, 1.80), *p* = 0.14). Among individual medications, only reported use of dexmedetomidine was associated with estimated ED occurrence, specifically, an 8.5% larger estimated ED occurrence (RD = 8.5%, OR (95% CI) = 1.54 (1.06, 2.22), *p* = 0.02). Among combined care strategy measures, there were no associations of “*similar* medication” variables with estimated ED occurrence. However, the “*exact* medication” and “routine screening plus *exact* medication” strategies were both significantly associated with lower percentage ED occurrence (*p* < 0.05 for both strategies). The “*exact* medication” strategy had 3.8% lower percentage ED occurrence (RD= -3.8%, OR (95% CI) = 0.37 (0.16, 0.84), *p* = 0.04) and the “routine screening plus *exact* medication” strategy had 3.4% lower percentage ED occurrence (RD=-3.4%, OR (95% CI) = 0.34 (0.14, 0.84), *p* = 0.02). Providers who estimated ED occurrence had significantly higher estimated numbers of surgeries per month (Estimated ED occurrence (*n* = 497): mean = 54.62, SD = 33.18; No estimated ED occurrence (*n* = 193): mean = 48.97, SD = 33.00; *p*=0.004) *[Data not shown in table]*.

### Adjusted models of association of location and care strategies with estimated ED occurrence (Table 5)

Associations between location and estimated ED occurrence while adjusting for variability in the number of surgeries and use of each care strategy were conducted as separate exploratory analyses. Each care strategy listed in Table 4 was modeled separately and adjusted model results are presented in Table 5. Interpretations of each model are described below.

**Table 5:** Multivariable modeling of the association of estimated emergence delirium occurrence with location after adjustment for reported care strategy and estimated number of surgeries per month

Model #	Care Strategy: Reported Use Yes compared to No (reference group)	Location. OR (95% CI), *p*-value: Non-PHS providers compared to PHS providers (reference group)	Care Strategy. OR (95% CI), *p*-value: Present compared to Absent (reference group)	Number of Surgeries. OR (95% CI), *p*-value: Continuous integer
1	Routine Screening	2.90 (1.42, 5.91) *p* = 0.003	1.39 (0.98, 1.96) *p* = 0.06	1.00 (1.00, 1.01) *p* = 0.07
2	Midazolam	3.01 (1.44, 6.27) *p* = 0.003	0.79 (0.55, 1.14) *p* = 0.22	1.00 (1.00, 1.01) *p* = 0.07
3	Volatile Anesthetic	2.65 (1.30, 5.38) *p* = 0.007	0.96 (0.66, 1.40) *p* = 0.84	1.00 (1.00, 1.01) *p* = 0.07
4	Ketamine	2.71 (1.33, 5.53) *p* = 0.006	1.12 (0.80, 1.58) *p* = 0.51	1.00 (1.00, 1.01) *p* = 0.07
5	Dexmedetomidine	2.98 (1.47, 6.03) *p* = 0.002	1.65 (1.14, 2.40) *p* = 0.009	1.00 (1.00, 1.01) *p* = 0.07
6	Propofol	2.65 (1.31, 5.36) *p* = 0.006	1.20 (0.77, 1.87) *p* = 0.43	1.00 (1.00, 1.01) *p* = 0.07
7	*Similar* Medication Strategy	2.76 (1.34, 5.72) *p* = 0.006	1.14 (0.71, 1.82) *p* = 0.58	1.00 (1.00, 1.01) *p* = 0.07
8	Routine Screening plus *Similar* Medication Strategy	2.83 (1.35, 5.94) *p* = 0.006	1.20 (0.69, 2.12) *p* = 0.52	1.00 (1.00, 1.01) *p* = 0.07
9	*Exact* Medication Strategy	2.13 (0.95, 4.79) *p* = 0.07	0.62 (0.24, 1.62) *p* = 0.33	1.00 (1.00, 1.01) *p* = 0.08
10	Routine Screening plus *Exact* Medication Strategy	2.16 (0.97, 4.82) *p* = 0.06	0.60 (0.21, 1.74) *p* = 0.34	1.00 (1.00, 1.01) *p* = 0.08

Ten separate models were analyzed using all available data (*N* = 690). Odds Ratio (OR) values indicate the relative odds of estimated ED occurrence. An OR value over 1.00 means that the non-reference group has greater odds of ED occurrence compared to the reference group. When the 95% Wald confidence interval (CI) around the OR does not include an OR of 1.00, that means the association with having estimated ED occurrence is statistically significant. For example, the results of Model # 10, “Routine Screening plus *Exact* Medication Strategy,” is interpreted as follows: Location: Compared to providers at PHS, non-PHS providers had 2.16 times greater odds of having estimated ED occurrence (a statistically non-significant finding); Care Strategy: a provider reporting that they used the “Routine Screening plus *Exact* Medication Strategy” was associated with a 0.4 (= 1.0–0.6) lower odds of having estimated ED occurrence (a statistically non-significant finding); and Number of Surgeries: there was an equivalent association of number of surgeries with a provider having estimated ED occurrence (OR = 1.0, a statistically non-significant finding)

#### Routine screening (Model #1) 

The odds of having estimated ED occurrence increased by a factor of 2.90 among non-PHS compared to PHS providers (*p* = 0.003), even after adjusting for number of surgeries per month (OR = 1.0, *p* = 0.07) and routine screening (OR = 1.39, *p* = 0.06). Neither routine screening nor number of surgeries per month were associated with estimated ED occurrence in adjusted models as indicated by OR 95% CI that do not include 1.0 and p-values ≥ 0.05.

#### Use of individual medications (Models #2–6) 

Individual medications reported as being used were assessed with five separate logistic regression models (Table 5). For each medication, estimated ED occurrence was approximately 3-times greater among non-PHS than PHS providers (*p* < 0.01 for all). Individual medications assessed were not associated with estimated ED occurrence in adjusted models (*p* ≥ 0.05), except for dexmedetomidine (OR = 1.65, *p* = 0.009). This means that providers who estimated ED occurrence were 1.65-fold more likely to report that they used dexmedetomidine (*p* = 0.009) and 2.98-fold more likely to be a non-PHS provider (OR = 2.98, *p* = 0.002), even after adjusting for the number of surgeries per month (OR = 1.0, *p* = 0.07). Number of surgeries per month were not associated with estimated ED occurrence in adjusted modeling of any individual medications (approximate OR = 1.0, *p* ≥ 0.05 for all).

#### Use of the “*similar* medication” strategy (Model #7)

Estimated ED occurrence was increased by a factor of 2.76 among non-PHS compared to PHS providers (*p* = 0.006) after adjusting for number of surgeries per month (OR = 1.0, *p* = 0.07) and “*similar* medication” strategy (OR = 1.14, *p* = 0.58).

#### Use of the “routine screening plus *similar* medication” strategy (Model #8)

The combined “routine screening plus *similar* medication” strategy variable was associated with a 2.83 higher odds of estimated ED occurrence among non-PHS providers compared to PHS providers (*p* = 0.006) after adjusting for number of surgeries per month (OR = 1.0, *p* = 0.07) and the “routine screening plus *similar* medication” strategy (OR = 1.20, *p* = 0.52).

#### Use of the “*exact* medication” strategy (Model #9)

For the “*exact* medication*”* strategy model, neither location (OR = 2.13, p = 0.07), number of surgeries per month (OR = 1.0, p = 0.07), nor the “*exact* medication” strategy (OR=0.62, p = 0.33) were associated with estimated ED occurrence.

#### Use of the “routine screening plus *exact* medication” strategy (Model #10)

In the “routine screening plus *exact* medication” strategy model, estimated ED occurrence was not associated with location (OR = 2.16, *p* = 0.06), number of surgeries per month (OR = 1.0, *p* = 0.08), nor “routine screening plus *exact* medication” strategy (OR=0.60, *p* = 0.34).

### Adjusted models of association of care strategies with estimated ED occurrence [*Data not shown in table*]

When location was removed from the adjusted model, there were no overall associations between estimated ED occurrence and reported use of the following strategies when analyzed individually: routine screening (OR (95% CI) = 1.28 (0.92, 1.80), *p* = 0.15), midazolam (OR (95% CI) = 0.93 (0.66, 1.31), *p*=0.68), volatile anesthetics (OR (95% CI) = 1.05 (0.73, 1.52), *p*=0.78), ketamine (OR (95% CI) = 1.04 (0.74, 1.45), *p* = 0.82), or propofol (OR (95% CI) = 1.16 (0.74, 1.81), *p* = 0.51). The OR (95% CI) values for number of surgeries per month for each non-significant individual care strategy was 1.01 (1.00, 1.01) (~ *p* = 0.05 for all). Interestingly, there was an increase in estimated ED occurrence associated with the reported use of dexmedetomidine (OR (95% CI) = 1.53 (1.06, 2.21), *p* = 0.025; Number of surgeries per month OR (95% CI) = 1.01 (1.00, 1.01), *p* = 0.05).

For the models of the combined care strategies of “*similar* medication” with and without “routine screening,” there were no associations of estimated ED occurrence with: “*similar* medication” (OR (95% CI) = 0.96 (0.62, 1.49), *p* = 0.86), or “routine screening plus *similar* medication” (OR (95% CI) = 0.94 (0.57, 1.61), *p* = 0.83). The OR (95% CI) values for number of surgeries per month for each non-significant “*similar* medication” care strategy was 1.01 (1.00, 1.01) (*p* = 0.05 for both). However, for the “*exact* medication” with and without “routine screening,” there was a decreased association between estimated ED occurrence and both the “*exact* medication” (OR (95% CI) = 0.40 (0.17, 0.90), *p* = 0.03; Number of surgeries per month OR (95% CI) = 1.01 (1.00, 1.01), *p* = 0.06) and “routine screening plus *exact* medication” strategies (OR (95% CI) = 0.36 (0.14, 0.92), *p* = 0.03; Number of surgeries per month OR (95% CI) = 1.01 (1.00, 1.01), *p* = 0.07).

## Discussion

Previous literature noted a considerable need for more research on ED prevention and treatment through specific and concurrent care practices, such as pre-screening all patients for risk of ED and use of medications associated with lowering the risk of ED (Souhayl Dahmani et al. 2012; S.-J. Lee and Sung 2020; Nguyen et al. 2016; Taylor and Pileggi 2021; Taylor et al. 2022b; Tolly et al. 2021). Huang et al. conducted a review of literature about anesthetic choices for management of ED and found that the multifactorial nature of ED translated into mixed results about the anesthetic medications associated with a lower risk of ED (Huang et al. 2023). Additionally, they found that the infrequent inclusion of some care strategies across the studies prevented their ability to properly evaluate the effect of concurrent multidisciplinary care strategies on ED. Because of the cross-sectional survey design, the analytic results were interpreted as associations, not as predictive, causal, or time-based relationships. However, as a strength, our survey included measurement of reported use of multiple care strategies concurrently.

We hypothesized that PHS providers would have lower percentage estimated ED compared to providers at non-PHS facilities. Our findings support our hypothesis and show that PHS providers (50%) had significantly lower percentage estimated ED compared to non-PHS providers (73.2%) in bivariate analyses (OR = 2.73, *p* = 0.005). We also found significantly lower estimated ED occurrence in multivariable adjusted analyses when adjusting for all care strategies (OR ~ = 3, *p* < 0.01 for all) and number of surgeries per month (OR = 1.00, *p* = 0.07 for all), except for the “*exact* medication” and “routine screening plus *exact* medication” care strategies (*p* > 0.05 for both).

Further we hypothesized that the “*exact* medication” care strategies would be associated with lower percentage estimated ED regardless of location. We found that the “*exact* medication” care strategies were associated with lower percentage estimated ED regardless of location in bivariate analyses (“*exact* medication”: OR = 0.37, *p* = 0.04; “screening plus *exact* medication”: OR = 0.34, *p* = 0.02) and multivariable adjusted analyses (“*exact* medication”: OR = 0.40, *p* = 0.03; “screening plus *exact* medication”: OR = 0.36, *p* = 0.03), which included number of surgeries per month. The similarity in bivariate and adjusted OR results indicate that number of surgeries per month had no substantive effect on the association between “*exact* medication” care strategies and estimated ED occurrence. Small sample sizes of the combined “*exact* medication” care strategy variables in the current project likely hinder our ability to assess interactions in adjusted modeling, but these results could be hypothesis generating for future research.

In the multivariable models of all individual medication care strategies that did not include location, we found that providers who estimated ED occurrence had 1.53-fold greater odds of reported use of dexmedetomidine. This result was not expected because much of the prior literature reported that preoperative and/or intraoperative use of dexmedetomidine was associated with a lower rate of ED (Bartoszek et al. 2021; Dahmani et al. 2010; Hauber et al. 2015; Hu et al. 2021; Kim et al. 2019; H. S. Lee et al. 2018; Nguyen et al. 2016; Ni et al. 2015; Pasin et al. 2015; Rao et al. 2020; Sethi et al. 2014; Shukry et al. 2005; Sun et al. 2022; Taylor et al. 2022a; Wang et al. 2016; Zhang et al. 2019). It is possible that providers who reported use of dexmedetomidine used it to prevent or, conversely, treat an episode of ED. Because these analyses could not account for conditions of use, such as dosing or timing of administration, the authors could not evaluate the underlying reason for medication administration, which we suggest accounting for in future research. No other individual medication care strategies were associated with estimated ED occurrence.

When we explored the “*exact* medication*”* strategy with inclusion of location, we found in bivariate unadjusted analyses that 42.7% more providers at PHS reported using this strategy than non-PHS (*p* < 0.001). Additionally, the “*exact* medication” strategy was associated with 3.8% lower percentage ED occurrence in bivariate analyses. Multivariable adjusted logistic regression modeling of the “*exact* medication*”* strategy (Model #9) suggested comparable but not statistically significant results, perhaps due to the small sample sizes associated with “*exact* medication” care strategies. Based upon the divergent statistical significance between unadjusted bivariate (Table 4) and adjusted modeling associations (Table 5), we measured the potential effect of low sample sizes of providers who estimated ED occurrence by location by “*exact* medication” strategy. A total of 6 of 9 non-PHS providers (67%) reported using the “*exact* medication” strategy compared to 6 of 15 PHS providers (40%); a 27% RD between locations. *[Data not shown in tables]* Given the clinical significance associated with the magnitude of this RD and that statistical significance was reached for smaller effect sizes with larger sample sizes in this project, the lack of statistical significance in Model #9 is potentially due to inadequate sample size.

Exploration of the “routine screening plus *exact* medication*”* strategy found in bivariate unadjusted analyses that 37.3% more providers at PHS reported using the “routine screening plus *exact* medication” strategy than non-PHS providers (p < 0.001). Additionally, the “routine screening plus *exact* medication” strategy was associated with 3.4% lower percentage ED occurrence in bivariate analyses. Like the “*exact* medication” strategy variable (Model #9), we assessed the potential effect of low sample sizes on results from analyses related to estimated ED occurrence by location by the “routine screening plus *exact* medication” strategy. Among providers who reported using the “routine screening plus *exact* medication” strategy, a total of 3 of the 6 (50%) non-PHS providers and 6 of the 13 (46%) PHS providers reported estimated ED occurrence. *[Data not shown in table]* The small sample sizes in the adjusted model of the “routine screening plus *exact* medication” strategy (Model #10), just as with the “*exact* medication” strategy (Model #9), may be affecting our ability to detect statistical significance.

As discussed above, results from the current analyses suggest that the application of care strategies recommended in the PHS intervention may be hypothesis generating in future evaluations. These findings indicate potential patterns and associations that warrant further investigation in more rigorously controlled studies. Future research should aim to validate these preliminary observations and determine their applicability across broader populations and settings.

### Limitations and future research

As an alternative to collecting data through a survey, PHS staff first explored the use of medical records and patient safety reports as retrospective data sources. However, those sources were insufficient due to the infrequent and irregular recording of ED-related information. Unfortunately, this type of deficiency in data is not uncommon when studying ED. Others have reported challenges in conducting ED research due to a lack of standard or agreed upon diagnostic codes or tools, (Bartoszek et al. 2021; Dahmani et al. 2010; Greiner and Kremer 2019; Hauber et al. 2015; Kuratani and Oi 2008; S.-J. Lee and Sung 2020; McGuire 2012; Rao et al. 2020; Tolly et al. 2021) terminology, (Dahmani et al. 2010; Greiner and Kremer 2019; Hauber et al. 2015; S.-J. Lee and Sung 2020; Menser and Smith 2020; Tolly et al. 2021; Wang et al. 2023; Yuan et al. 2022) and risk factors, (Bartoszek et al. 2021; Souhayl Dahmani et al. 2012; S.-J. Lee and Sung 2020; McGuire 2012; McGuire and Burkard 2010; Taylor and Pileggi 2021; Taylor et al. 2022a, b; Tolly et al. 2021; Urits et al. 2020; Viswanath et al. 2015; Zhang et al. 2019) all of which can create challenges when using medical records and safety reports as data sources.

Due to the retrospective, anonymous, cross-sectional, self-report survey design used in this project, there was potential for several limitations, including recall bias (retrospective data collection), response bias (self-selection), potential for repeat responses and inability to validate responses (reliance upon anonymous self-report only), inability to directly measure the effect of clinical activities and interventions on ED at PHS and non-PHS locations, and failure to assess causal relationships (cross-sectional design). Lastly, because we used a novel unvalidated survey, respondent confusion was possible and could have caused issues with internal and external validity. For future work related to ED, we recommend using a prospective design with objective repeated measurements that account for a greater breadth of potential covariates and is conducted as a component analysis (Cooper et al. 2020; Sutcliffe et al. 2015; Ward-Horner and Sturmey 2010) to identify which intervention components are necessary to achieve the desired quality of care and safety.

## Conclusions

We hypothesized that providers from the PHS location would be less likely to report having estimated ED occurrence compared to providers at the non-PHS location and the reported care strategies promoted in the PHS intervention (Taylor et al. 2022a, b) would be associated with lower percentage estimated ED regardless of location. Four separate aims were created to support testing of the hypothesis. Aim 1 evaluated differences in reported use of screening and medication care strategies between locations. Non-PHS providers were less likely to report routine patient screening for ED risk factors, more likely to report use of midazolam and volatile anesthetics among patients with PTSD, and less likely to report use of dexmedetomidine and ketamine among patients with PTSD. Aim 2 evaluated providers’ estimates of ED occurrence by location. Non-PHS providers were more likely to report estimated ED occurrence when compared to providers at PHS. Aim 3 assessed associations, regardless of location, between occurrence of estimated ED and use of routine screening and medication strategies. Lower estimated ED occurrence was associated with greater reported estimated use of the “*exact* medication” strategy. Aim 4 evaluated the differences in estimated ED occurrence between PHS and non-PHS locations while adjusting for each care strategy and number of surgeries per month. Analyses supported the hypothesis that providers at PHS were less likely to have estimated ED occurrence even after statistical adjustment for care strategies and number of surgeries per month.

Despite the limitations of utilizing a cross-sectional self-report survey, this project can provide a useful descriptive contribution to the literature on ED practice variation. Collectively, these analyses found that fewer non-PHS providers reported using the patient care strategies promoted by the PHS intervention than PHS providers and were more likely to have estimated ED occurrence. While we acknowledge that this analysis would be best used to generate new hypotheses for more rigorous study, we would recommend in future work the use of concurrent prospective measurements of screening and medication use to move the field forward with more stringency and clarity than has been conducted in the past. Such efforts will be critical to establishing more definitive links between provider practices and patient outcomes.

## Data Availability

The datasets used and/or analyzed during the current study are available from the corresponding author on reasonable request.
